# Recent Advances in Studies on the Therapeutic Potential of Dietary Carotenoids in Neurodegenerative Diseases

**DOI:** 10.1155/2018/4120458

**Published:** 2018-04-16

**Authors:** Kyoung Sang Cho, Myeongcheol Shin, Sunhong Kim, Sung Bae Lee

**Affiliations:** ^1^Department of Biological Sciences, Konkuk University, Seoul 05029, Republic of Korea; ^2^Disease Target Structure Research Center, Korea Research Institute of Bioscience and Biotechnology, Daejeon 34141, Republic of Korea; ^3^Department of Bioscience, University of Science and Technology, Daejeon 34113, Republic of Korea; ^4^Department of Brain and Cognitive Sciences, DGIST, Daegu 42988, Republic of Korea

## Abstract

Carotenoids, symmetrical tetraterpenes with a linear C40 hydrocarbon backbone, are natural pigment molecules produced by plants, algae, and fungi. Carotenoids have important functions in the organisms (including animals) that obtain them from food. Due to their characteristic structure, carotenoids have bioactive properties, such as antioxidant, anti-inflammatory, and autophagy-modulatory activities. Given the protective function of carotenoids, their levels in the human body have been significantly associated with the treatment and prevention of various diseases, including neurodegenerative diseases. In this paper, we review the latest studies on the effects of carotenoids on neurodegenerative diseases in humans. Furthermore, animal and cellular model studies on the beneficial effects of carotenoids on neurodegeneration are also reviewed. Finally, we discuss the possible mechanisms and limitations of carotenoids in the treatment and prevention of neurological diseases.

## 1. Introduction

Carotenoids are natural pigments present in various organisms, such as plants, animals, and microorganisms. For example, the orange color of carrots and the red color of tomatoes are due to their carotenoid components [1]. Plant, algae, and fungi produce >600 different types of carotenoids. Animals obtain carotenoids from food since they cannot synthesize them. As pigment molecules, carotenoids play a role in the process of photosynthesis, either in photoprotection or light collection [2]. Carotenoids confer photoprotection by dissipating light energy that is not used directly for photosynthesis, and they contribute to photosynthesis through light collection, during which they pass light through to the chloroplast [2]. Carotenoids also act as antioxidants that reduce reactive by-products, such as reactive oxygen species (ROS), during photosynthesis [3]. As a result, carotenoids protect the photosynthetic apparatus from oxidative damage. In addition, carotenoids play various other roles in nature, including the development and oxidative stress signaling in plants, sex-related coloration patterns, and as a precursor for vitamin A in many species [3, 4].

Carotenoids, also known as tetraterpenoids, are C40 hydrocarbons that have isoprenoids as building units (Table 1). The C40 carbon skeleton of carotenoids is produced by the linkage of two C20 geranylgeranyl diphosphate molecules; all of the carotenoid variants are derived from the skeleton [4]. Carotenoids can be divided into two groups according to their polarity: xanthophylls (polar carotenoids such as astaxanthin, *β*-cryptoxanthin, lutein, and zeaxanthin) and carotene (nonpolar carotenoids such as *α*-carotene, *β*-carotene, and lycopene) [5]. The distinctive structural feature of carotenoids is the long, alternating double and single bond system, which is associated with light absorption and oxidation [4].

The major sources of carotenoids in the human diet are fruits and vegetables, which have various colors, such as green, red, orange, and yellow [6]. Humans consume approximately 40 carotenoids from common fruits and vegetables (Table 1) [7]. Dark green vegetables, such as broccoli, coriander, kale, and spinach, contain a large number of chloroplasts, in which most carotenoids exist; therefore, they possess high concentrations of carotenoids [8]. As chloroplasts generally contain the most consistent carotenoid composition [9], the distribution of carotenoids is similar among different plant species in this group [7]. On the other hand, in red-, orange-, or yellow-colored fruits and vegetables, carotenoids are mainly accumulated in chromoplasts, which are usually converted from chloroplasts during ripening [10]. As chromoplasts in different plant species contain various carotenoids, the carotenoid distribution in this group is diverse [6]. Some seafood and animal foods also contain carotenoids. Animals cannot synthesize carotenoids; instead, they ingest carotenoids through foods and accumulate these molecules in their bodies. As a result, some animal foods contain carotenoids. For example, high concentrations of lutein and zeaxanthin accumulate in egg yolks [11]. Milk and dairy products, salmonid fish, and crustaceans also provide various carotenoids [12]. The main carotenoid in bovine milk is *β*-carotene [13], whereas the major carotenoids in salmonid fish and crustaceans are astaxanthin and canthaxanthin [12, 14, 15]. In addition, some edible brown seaweeds contain fucoxanthin as a major carotenoid [16, 17].

Carotenoids are differentially distributed in various organs of the human body. Interestingly, xanthophylls account for 66–77% of the total carotenoids in the frontal and occipital lobes of the human brain [18], whereas less than 40% of the total carotenoids in most tissues and plasma are reported to be xanthophylls [19–21]. It was reported that the human brain contains sixteen carotenoids, with the major carotenoids being anhydrolutein, *α*-carotene, *α*-cryptoxanthin, *cis*- and *trans*-*β*-carotene, *β*-cryptoxanthin, lutein, *cis*- and *trans*-lycopene, and zeaxanthin [18]. Given their property of protecting tissues from oxidative stress and their localization in the brain, the role of carotenoids in preventing or treating oxidative stress-associated diseases, including neurodegenerative diseases, is of interest.

As carotenoids have various physiological activities, such as antioxidant activity, the amount of carotenoid in the human body is important for health. Therefore, the intake of carotenoids through the diet is associated with the prevention and treatment of various diseases, including age-related macular degeneration [22], cancer [23, 24], cardiovascular diseases [25], and neurodegenerative diseases [5]. In the present paper, we review the latest studies that show the effects of dietary carotenoids on neurodegenerative diseases, and discuss the prospect of the use of carotenoids in the prevention and treatment of these diseases.

## 2. Bioactivities of Carotenoids

As stated above, most carotenoids have a symmetrical tetraterpene structure with a linear C40 hydrocarbon backbone (Table 1). These highly unsaturated fatty chains are susceptible to modifications, such as *cis*-*trans* isomerization or cyclization, and result in the characteristic coloration induced by light absorption. Owing to their highly lipophilic structures, carotenoids are found in the lipid membrane. Nonpolar carotenes reside in the inner part of the membrane, whereas polar xanthophylls are located across the bilayer, tilted ~40° from the axis normal to the membrane plane [26, 27]. Inserted carotenoids may affect the physical properties of the lipid bilayer; however, their exact function in the membrane remains unclear besides in their prevention of the oxidative damage of lipids [27]. Significant evidence has shown that carotenoids can reduce oxidative damage by scavenging ROS and exert anti-inflammatory effects *in vivo* (Table 2) [28].

### 2.1. Antioxidant Activity

Carotenoids have been demonstrated to be one of the most potent natural singlet oxygen scavengers, with a fast quenching rate (≅10^10^ M^−1^ s^−1^) [29]. They can effectively neutralize ROS and other free radicals to provide protection against oxidation in both photosynthetic and nonphotosynthetic organisms [6, 16, 29–37]. However, each carotenoid shows different antioxidant activities, owing to the presence of functional groups with increasing polarities as well as the number of conjugated double bonds [31]. The antioxidant property of carotenoids has inspired many epidemiological and clinical studies that have investigated if these pigment molecules are able to prevent various ROS-mediated disorders such as cancer, inflammation, retinal degeneration, and neurodegeneration. In the case of cancer, many studies have shown that carotenoid consumption is correlated with a reduced risk of several types of cancer; however, other studies have shown that the cancer-preventive effects of carotenoids are negligible or even that they are carcinogenic [38, 39].

Lutein is a xanthophyll and the most abundant carotenoid in the human retina and brain [18, 40]. The Age-Related Eye Disease Study (AREDS) showed that a formulation consisting of vitamins C, E, *β*-carotene, and zinc is beneficial for the prevention of age-related macular degeneration (AMD) [41]. In a second study, although primary analysis from the AREDS2 did not reveal a benefit of daily supplementation with lutein/zeaxanthin on AMD progression [42], secondary exploratory analyses suggested that lutein/zeaxanthin were helpful in reducing this risk [43]. In addition, given that increased oxidative stress and inflammation are observed in age-related macular degeneration [44], lutein supplementation may improve visual function through antioxidant activity.

In addition to their antioxidant activities, carotenoids can protect cells from the oxidative stress induced by some stressors via activation of endogenous antioxidant enzymatic activities and a reduction in DNA damage. Crocetin, a pharmacologically active metabolite of *Crocus sativus* L., exerts cardioprotective effects by increasing superoxide dismutase (SOD) and glutathione peroxidase activities in cardiac hypertrophy induced by norepinephrine in rats [35]. Crocin, another component of *Crocus sativus* L., has also been shown to increase SOD activity to prevent the death of PC-12 cells during serum/glucose deprivation [34]. Recent studies have demonstrated that marine carotenoids such as astaxanthin and fucoxanthin also display antioxidant properties by activating the antioxidant network, including SOD and catalase [45, 46]. In addition, *β*-cryptoxanthin protects human cells from H_2_O_2_-induced damage by stimulating the repair of damage caused by DNA oxidation as well as by its antioxidant activity [36]. Lycopene and *β*-carotene also provide protection against DNA damage at low concentrations [32]. However, opposite effects have been shown at higher concentrations in cells with oxidative damage [32].

### 2.2. Antineuroinflammation

Neuroinflammation is a local response of the nervous system during neurodegeneration, trauma, and autoimmune disorders. A variety of cell types, including astrocytes, microglia, vascular cells, neutrophils, and macrophages, are involved in neuroinflammation [47]. Growing evidence suggests that neuroinflammation is one of the pathological features of many neurodegenerative disorders and autoimmune diseases, such as multiple sclerosis [44, 47, 48]. In the last decade, some carotenoids have been shown to have antineuroinflammatory effects *in vivo*. Among the polar xanthophylls, the ability of lutein to suppress inflammation has been demonstrated in murine retinal cells [49–51] and in a clinical trial studying retinal health in preterm infants [52]. In addition, it has been shown that lutein reduces lipid peroxidation and proinflammatory cytokine release by suppressing the activation of the nuclear factor-*κ*B (NF-*κ*B) pathway in the presence of a variety of oxidative stressors [53–56]. It has also been demonstrated that crocin and crocetin are able to suppress the production of proinflammatory cytokines and nitric oxide by lipopolysaccharide, interferon *γ*, and *β*-amyloid (A*β*) stimulation in microglial cells [57]. Astaxanthin, a member of the xanthophyll family that confers the pink color in flamingos, has an anti-inflammatory effect and antioxidant activity similar to other carotenoids [58, 59]. Furthermore, astaxanthin has also been found to reduce hippocampal and retinal inflammation in streptozotocin-induced diabetic rats, alleviating cognitive deficits, retinal oxidative stress, and depression [60–62]. Fucoxanthin, another member of the marine xanthophylls, exerts anti-inflammatory effects against various stimuli through Akt, NF-*κ*B, and mitogen-activated protein kinase pathways [63].

Lycopene, one of the carotenes present in large amounts in tomatoes, has been demonstrated to reduce neuroinflammatory phenotypes, depression-like behaviors, and inflammation-induced cognitive function defects in murine models [64–66]. As a whole, cellular and animal models have revealed that carotenoids are potent anti-inflammatory agents in the nervous system and act through the suppression of inflammation pathways.

### 2.3. Modulation of Autophagy

Autophagy, a catabolic process necessary for the cleanup of damaged organelles, protein complexes, and even single proteins, as well as for the recycling of nutritional building blocks, has been implicated in numerous disorders and conditions such as aging, cancer, and neurodegeneration. A growing amount of evidence strongly suggests that autophagy removes misfolded or aggregated proteins, the main features of most neurodegenerative diseases, for example, tau fibrils in Alzheimer's disease (AD) and Lewy bodies in Parkinson's disease (PD) [67]. Recent studies have shown that some carotenoids are able to modulate autophagy in cellular and animal models. It has been recently demonstrated that lutein attenuates cobalt chloride-induced autophagy via the mTOR pathway in rat Müller cells [68], whereas it induces autophagy through the upregulation of Beclin-1 in retinal pigment epithelial cells [69, 70]. Crocin has also been shown to have a paradoxical effect on autophagy. The induction of autophagy by crocin occurs during hypoxia, and the inhibition of autophagy by crocin occurs during reperfusion [71]. Lycopene has also been shown to be involved in autophagy [72–74]. Astaxanthin has been found to attenuate autophagy in hepatic cells [75, 76]. In a model of murine traumatic brain injury, fucoxanthin has the ability to protect neuronal cells from death through the activation of autophagy and the nuclear factor erythroid 2-related factor pathway [77]. The modulation of autophagy by carotenoids remains a controversial topic, and the precise molecular mechanism of this modulation remains unclear.

## 3. Beneficial Effects of Carotenoids on Neurodegenerative Diseases

### 3.1. Neurodegenerative Diseases

Neurodegenerative diseases are neuronal disorders that feature a progressive loss of neurons and are associated with protein aggregates [78]. The major neurodegenerative diseases include AD, PD, Huntington's disease (HD), and amyotrophic lateral sclerosis (ALS), all of which have disease-specific causative factors and pathological features. For examples, senile plaques with A*β* aggregates and fibrillary tangles with hyperphosphorylated tau are hallmarks of AD [79]. Similarly, aggregation of *α*-synuclein, huntingtin, and TAR DNA-binding protein 43 is associated with PD, HD, and ALS, respectively [80–82].

Although these neurodegenerative diseases have different causative factors, they share common features that might be closely related to the onset and progress of disease by the induction of neuronal cell death. One of the shared features is oxidative stress due to elevated ROS production during disease progression [78]. ROS are reactive chemicals with oxygen that can attack and damage the macromolecules, such as lipids, DNA, and proteins, of living cells [83]. In neuronal cells of patients with neurodegenerative diseases, ROS levels are increased by various cellular events, including mitochondrial insults and release of redox metals that interact with oxygen [84, 85], which result in neuronal cell death [85]. In addition, the pathological environment of neurodegenerative diseases, such as the increase in protein aggregates, results in sustained inflammation due to microglia activation [86]. Although the inducers of inflammation vary among different diseases, chronic inflammation is induced in neurons through largely common mechanisms [87]. Once neuroinflammation is chronically activated, cytokines and chemokines are released by long-standing activated microglia and oxidative stress is increased, which may be detrimental to neurons [88].

Given that oxidative damage and increased neuroinflammation are critically related with the pathogenesis of and late-onset massive neuronal loss in neurodegenerative diseases, the neuroprotective effect of carotenoids has been of specific interest in the search for effective treatments for these diseases. To provide an update on the latest advances in this field, we have reviewed the papers published in recent years in the following paragraphs.

### 3.2. Animal and Cellular Model Studies on the Beneficial Effects of Carotenoids on Neurodegenerative Diseases

Controlled animal model or cell culture studies allow for the accurate assessment of the sole influence of carotenoid administration on neurodegenerative diseases that human studies do not. Indeed, numerous experimental studies have recently highlighted the beneficial effects of carotenoids on neurodegenerative diseases (Table 2). Notably, most of these recent experimental studies have focused on either AD or PD.

In the case of well-studied lycopene, administration of lycopene in murine models of AD leads to the attenuation of mitochondrial oxidative damage [89] and inhibition of NF-*κ*B activity and related expression of proinflammatory cytokines in the brain [64], which together may contribute to the suppression of A*β* formation [90] and improvement of memory retention [64, 89]. In a recent study that used a tau transgenic mouse model for AD, dietary lycopene supplementation was shown to improve cognitive performance [91]. Similarly, in the context of PD, lycopene-rich tomato powder intake successfully prevented the decline in striatal dopamine levels and degeneration of nigral dopaminergic neurons in rodent models of PD [92, 93]. Consistently, in more recent studies, administration of lycopene was shown to protect against rotenone-induced oxidative stress, neurobehavioral impairments [94], and depletion of dopamine and its metabolites by 1-methyl-4-phenyl-1,2,3,6-tetrahydropyridine [95]. Moreover, the effect of lycopene was also experimentally assessed *in vivo* in the context of HD. Administration of lycopene alone [96–98], in combination with epigallocatechin-3-gallate [96], or with quercetin and poloxamer 188 [99] showed protective effects against HD-like symptoms induced by 3-nitropropionic acid in rodent models. Consistent with the results obtained from animal model studies, lycopene treatment was also recently shown to be very effective in attenuating neuropathic phenotypes in cultured cell models of AD [100–102] and PD [103]. Interestingly, the beneficial effects of lycopene were also confirmed in a study using a *Caenorhabditis elegans* model for AD [104].

In addition to lycopene, other dietary carotenoids such as fucoxanthin, astaxanthin, crocin, and crocetin have recently begun to be investigated experimentally for their potential beneficial effects. The beneficial effect of fucoxanthin was recently assessed in the context of AD [105, 106]. Moreover, astaxanthin has been shown to protect neurons in the context of various neurodegenerative diseases, including AD [107, 108], PD [109–112], and ALS [113]. Similarly, crocin was recently shown to be beneficial in both AD [114, 115] and PD [116–119]. Other recent studies on crocetin also support the beneficial effects of carotenoids on AD [120–122]. Of note, Tiribuzi et al. [122] used monocytes derived from patients with AD for analysis, and concluded that *trans*-crocetin improved the clearance of A*β in vitro* through the involvement of cathepsin B.

### 3.3. Human Studies on the Beneficial Effects of Carotenoids on Neurodegenerative Diseases

Consistent with the results obtained from animal and cell culture model studies showing the beneficial effects of carotenoid treatment on neurodegenerative diseases, a number of epidemiological studies have also linked the consumption of a carotenoid-rich diet with a decreased risk of neurodegenerative diseases in humans (Table 2) [123–126].

The epidemiological correlation between disease risk and carotenoid intake (or its level in the blood) is evident in various neurodegenerative diseases (Table 2). In the case of AD, the most investigated neurodegenerative disease, several studies have reported lower concentrations of carotenoids such as *β*-carotene, lutein, and vitamin A in the blood plasma of AD patients than in control subjects [127–129]. A very recent case-control study showed that the concentration of six major carotenoids (*α*-carotene, *β*-carotene, *β*-cryptoxanthin, lutein, lycopene, and zeaxanthin) in serum was significantly lower in patients with AD than in cognitively normal control subjects [130]. The results of a study comparing the levels of plasma carotenoids between patients with AD and normal subjects suggested that maintaining a high level of lutein in relation to plasma lipids can reduce the risk of AD [131]. Consistently, Nolan et al. [132] concluded that the serum concentrations of lutein, zeaxanthin, and *meso*-zeaxanthin were significantly lower in AD patients than in control subjects. Similarly, high concentrations of lutein, lycopene, and zeaxanthin in the serum were associated with a lower risk of death from AD [133].

In addition to blood carotenoid levels, carotenoid intake has also been epidemiologically associated with a reduced risk of AD and decreased rates of cognitive decline [123–125]. Additional studies have suggested that there are potential beneficial effects of providing carotenoid supplementation to patients with AD [126]. Kiko et al. [134] and de Oliveira et al. [135] reported that the supply of antioxidant supplements, including astaxanthin and a vitamin complex containing *α*-tocopherol, ascorbic acid, and *β*-carotene, reduced A*β* contents in red blood cells and ROS generation in the cells of AD patients, respectively. Moreover, a potentially related result was also recently published in an elderly Chinese population, and showed carotenoids to be one of the most highly protective factors against mild cognitive impairment in a cross-sectional study based on a 33-item food frequency questionnaire collected from 2892 elderly subjects [136]. However, the sole influence of carotenoid consumption on the risk or progression of AD has not yet been clearly established in humans.

Unlike in AD, there have been inconsistencies on the association between carotenoid intake and the reduced risk of PD until very recently. Although a number of epidemiological studies have proposed a possible association between increased intake of both provitamin A and nonprovitamin A carotenoid species and the reduced risk of PD, the risk reduction was small and did not always reach statistical significance [6]. To clarify the inconsistencies observed in human studies, a recent systematic review that analyzed pooled data from relevant papers published between 1990 and 2013 raised the possibility that both *α*- and *β*-carotene levels might be inversely proportional to PD risk [137]. Following this systematic study, a very recent paper by Kim et al. [138] reported that the serum levels of some carotenoids (*α*-carotene, *β*-carotene, and lycopene) were significantly lower in PD patients, and that these carotenoids were inversely correlated with clinical variables representing disease progression. On the contrary, another study reported that the consumption of vitamin E and carotenoids was not associated with the risk of PD [139]. Thus, more studies are required to draw a solid conclusion on the relationship between carotenoid intake and PD risk reduction.

In the case of ALS, Fitzgerald et al. [140] analyzed pooled results from five published cohort studies on the association between carotenoids and ALS, and suggested that the ingestion of carotenoid-rich foods can prevent or delay the onset of ALS. Consistently, a recent paper published in *JAMA Neurology* [141] reported that a greater intake of antioxidant nutrients and foods high in carotenoids seems to be associated with more beneficial effects in ALS around the time of diagnosis. Lastly, in the case of HD, there have been no epidemiological studies on carotenoids published in recent years, unlike for the other representative neurodegenerative diseases described above.

Unlike animal model studies, the human studies conducted thus far have focused on the statistical assessment of the epidemiological correlation between the risk of disease and carotenoid intake (or its level in the blood), rather than clinical trials directly measuring the beneficial effects of carotenoid supplementation on the treatment of disease symptoms. However, as can be expected from the *in vivo* and *in vitro* bioactivities of carotenoids, a number of studies have shown that various carotenoids have beneficial effects on neurodegenerative diseases.

### 3.4. Possible Molecular Mechanisms of the Effects of Carotenoids on Neurodegenerative Diseases

Extensive studies suggest that carotenoids may inhibit the onset of neurodegenerative diseases through a variety of mechanisms. The effects of carotenoids have already been studied in different cellular contexts [28, 126, 142, 143] that may have the same working mechanisms as in neurodegenerative diseases. In the case of AD, it has been shown that, through ROS quenching, upregulation of antioxidant enzyme systems, hypocholesterolemic properties, antineuroinflammatory effects, antiamyloid aggregation activity, and regulation of amyloid oligomer-induced signaling, carotenoids may ameliorate mitochondrial dysfunction, oxidative stress, sustained neuroinflammation, impaired lipid metabolism, A*β* aggregation, and A*β* neurotoxicity, all of which are critically associated with the pathogenesis of AD [63, 105, 106, 144]. In neurodegenerative disease states, the various mechanisms of action of carotenoids are likely to occur simultaneously. For example, administration of lycopene resulted in the concomitant reduction of A*β*-induced mitochondrial dysfunction, inflammatory cytokine mediators, and caspase-3 activity in a rat model of AD [64]. Furthermore, astaxanthin treatment reduced A*β*-induced damage in a cultured cell model through several mechanisms including downregulation of apoptotic factors, inhibition of inflammatory cytokine mediating action, and simultaneous reduction of ROS [106].

How can a single substance exhibit these various effects? The functional diversity may be due to the strong antioxidant properties of carotenoids that regulate ROS, key regulators of various biological activities. ROS induces functional modification of macromolecules, including lipids, DNA, and proteins, in the aging brains and brains of patients with neurodegenerative diseases [78]. These modifications may affect cellular processes by altering gene expression and signal transduction [145]. For example, the oxidation of PTEN, a lipid phosphatase and suppressor of PI3-kinase pathway, via oxidative stress results in the activation of the NF-*κ*B pathway via I*κ*B kinase (IKK) activation [54]. Since NF-*κ*B regulates the expression of many genes, including oxidative stress-responsive and inflammation-related genes [146], a sustained increase in ROS in the brains of patients with neurodegenerative diseases may lead to an inflammatory response. Therefore, powerful antioxidants, such as carotenoids, can lower the level of ROS to mitigate cellular damage and simultaneously inhibit inflammatory responses by lowering the activity of NF-*κ*B. Indeed, recent studies have shown that various carotenoids suppress inflammation via inhibition of NF-*κ*B activity [56, 63, 147–152]. Given that oxidative stress and neuroinflammation are crucial to the onset and progress of various neurodegenerative diseases, it is expected that similar working mechanisms may be commonly applied to other neurodegenerative diseases.

In addition to these well-characterized cellular mechanisms of carotenoid functions, the recently proposed carotenoid-mediated regulation of autophagy described above also has a strong potential for protecting neurons in neurodegenerative disease conditions through the reduction of toxic disease proteins conferring neuronal toxicity. However, the role of autophagy in the effects of carotenoids on neurodegenerative diseases remains unclear. Further studies are needed to determine the role that carotenoid-regulated autophagy plays in neuroprotection.

## 4. Conclusion

In this article, we reviewed the recent updates on the beneficial effects of dietary carotenoids on neurodegenerative diseases. An increasing number of papers have demonstrated that dietary carotenoids protect neurons in the context of neurodegenerative diseases through several mechanisms, such as ROS quenching, upregulation of antioxidant enzyme systems, and antineuroinflammatory effects. Indeed, the number of research papers studying the link between carotenoids and neurodegenerative diseases has steadily increased to date. Notably, animal and cell culture model studies have recently begun to be actively conducted, and these model studies strongly support the hypothesis that carotenoid intake may have therapeutic potential in either preventing or ameliorating various neurodegenerative diseases. AD and PD have been more thoroughly studied in this regard than other types of rare neurodegenerative diseases (e.g., ALS and HD). In the rodent models of these diseases, administration of certain types of carotenoids, including lycopene, successfully attenuated not only cellular-level phenotypes such as mitochondrial oxidative damage and increased neuroinflammation, but also organism-level phenotypes such as memory impairment and locomotive defects. Of note, the beneficial effects of dietary carotenoids such as astaxanthin, crocin, crocetin, and fucoxanthin on neurodegenerative diseases have been recently studied in animal model systems, broadening our understanding of the association between carotenoid uptake and neurodegenerative diseases.

Although many of the studies presented in this paper demonstrate the beneficial effects of carotenoids as food nutrients on neurodegenerative diseases, some aspects of the carotenoid effect need to be clarified for medical use beyond food nutrients. First, the results of many studies have lacked an accurate analysis of the mechanisms by which carotenoids exert neuroprotective effects. The strong antioxidant properties of carotenoids can explain the neuroprotective effects of carotenoids in that one of the characteristic pathologies in neurodegenerative diseases is increased oxidative stress. However, the mechanisms by which carotenoids inhibit neuroinflammation and activate autophagy have not been thoroughly studied. In addition, many cell studies have shown that some carotenoids regulate the expression of antioxidant and inflammatory proteins, and it is interesting to note how they regulate gene expression. Second, clinical application studies in human patients are required. The causal relationship of the carotenoid effect in human patients can only be clarified by studies of this type. It may also be possible to infer the correlation between carotenoid intake and the onset of disease through comparative studies of races that eat different foods. Finally, in terms of the complexity in the pathogenic mechanisms underlying these diseases, it seems likely that simply increasing the dietary intake of carotenoids may exert only limited protective effects to neurons. For this reason, we expect that future studies determining other neuroprotective reagents/treatments that confer synergistic effects in combination with carotenoids in neurodegenerative diseases will be essential in finding effective treatments.

## Figures and Tables

**Table 1:** Representative food-derived carotenoids and their structures.

Carotenoid	Structure	Food source	Reference
*α*-Carotene	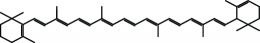	Banana, butternut, carrot, pumpkin	[11, 153–156]
*β*-Carotene	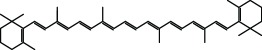	Apricots, banana, broccoli, cantaloupe, carrot, dairy products, honeydew, kale, mango, nectarine, peach, pumpkin, spinach, sweet potato, tomato	[1, 11, 13, 153, 154]
Crocetin	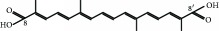	Gardenia fruit, saffron stigma	[157, 158]
Crocin	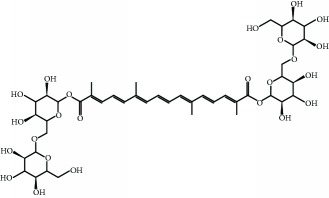	Gardenia fruit, saffron stigma	[159, 160]
*β*-Cryptoxanthin	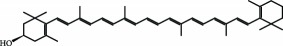	Apple, broccoli, celery, chili, crustaceans, grape, green beans, papaya, pea, peach, peppers, salmonid fish, squashes, tangerine	[11, 12, 14, 153, 161]
Lutein	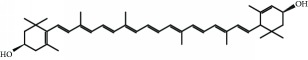	Apple, basil, broccoli, celery, crustaceans, cucumber, dairy products, grapes, green pepper, kale, kiwi, maize, parsley, pea, pumpkin, salmonid fish, spinach, squash	[1, 11, 12, 162, 163]
Lycopene	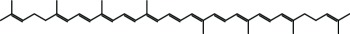	Grapefruit, guava, tomato, watermelon	[1, 11, 153, 164, 165]
Zeaxanthin	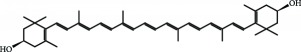	Basil, crustaceans, cucumber, dairy products, honeydew, kale, maize, mango, orange, parsley, salmonid fish, spinach	[1, 11, 12, 162]
*Marine*			
Astaxanthin	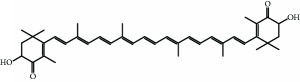	Crustaceans, algae, salmonid fish	[12, 14, 15, 36]
Fucoxanthin	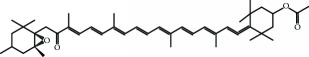	Brown seaweeds	[16, 17, 166]

**Table 2:** Bioactivities of representative food-derived carotenoids and their implications in neurodegenerative diseases.

Carotenoid	Bioactivity	Reference	Implication in ND	Reference
*α*-Carotene	Antioxidant	[6, 31]	AD: human	[130]
			PD: human	[137, 138]
*β*-Carotene	Antioxidant	[6, 30, 31]	AD: human	[130, 134, 135]
			PD: rodent	[137, 138]
			ALS: cell	[113]
Crocetin	Antioxidant	[35]	AD: cell	[121, 125]
	Antineuroinflammation	[54]		
Crocin	Antioxidant	[34]	AD: rodent	[115]
	Antineuroinflammation	[57]	Cell	[114]
	Autophagy	[71]	PD: rodent	[117, 118]
			*Drosophila*	[119]
			Cell	[116]
*β*-Cryptoxanthin	Antioxidant	[6, 36]	AD: human	[130]
Lutein	Antioxidant	[6, 31]	AD: human	[130–133]
	Antineuroinflammation	[49–51, 53]	PD: rodent	[167]
	Autophagy	[68–70]	HD: rodent	[168]
Lycopene	Antioxidant	[6, 31]	AD: human	[130, 133]
	Antineuroinflammation	[64, 66]	Rodent	[36, 89, 91]
	Autophagy	[72–74]	*C. elegans*	[104]
			Cell	[100–102]
			PD: human	[138]
			Rodent	[92–95]
			Cell	[103]
			HD: rodent	[96–99]
Zeaxanthin	Antioxidant	[6, 31]	AD: human	[132, 133]
*Marine*				
Astaxanthin	Antioxidant	[6, 31, 33]	AD: human	[134]
	Antineuroinflammation	[58, 59, 62]	Cell	[107, 108]
	Autophagy	[75, 76]	PD: rodent	[110]
			Cell	[109–112]
			ALS: cell	[113]
Fucoxanthin	Antioxidant	[16]	AD: rodent	[106]
	Antineuroinflammation	[63]	Cell	[105]
	Autophagy	[77]		

## Abbreviations

A*β*: *β*-Amyloid
AD: Alzheimer's disease
ALS: Amyotrophic lateral sclerosis
HD: Huntington's disease
PD: Parkinson's disease
ROS: Reactive oxygen species.
